# Measurement of Plasma Resistin Concentrations in Horses with Metabolic and Inflammatory Disorders

**DOI:** 10.3390/ani12010077

**Published:** 2021-12-30

**Authors:** Beatriz Fuentes-Romero, Alberto Muñoz-Prieto, José J. Cerón, María Martín-Cuervo, Manuel Iglesias-García, Escolástico Aguilera-Tejero, Elisa Díez-Castro

**Affiliations:** 1 Department of Equine Internal Medicine, University of Extremadura, 10004 Cáceres, Spain; mariamc@unex.es; 2 Interdisciplinary Laboratory of Clinical Pathology, Interlab-UMU, University of Murcia, 30003 Murcia, Spain; alberto.munoz@um.es (A.M.-P.); jjceron@um.es (J.J.C.); 3 Department of Equine Surgery, University of Extremadura, 10004 Cáceres, Spain; manuiglesiasgarcia@gmail.com; 4 Department of Equine Internal Medicine, University of Córdoba, 14014 Córdoba, Spain; pv1agtee@uco.es (E.A.-T.); elisadiez@uco.es (E.D.-C.)

**Keywords:** resistin, insulin dysregulation, adipokine, inflammation

## Abstract

**Simple Summary:**

Obesity and its associated complications, such as metabolic syndrome, are an increasing problem in both humans and horses in the developed world. Adipose tissue is a key endocrine organ that communicates with other organs by multiple endocrine substances called adipokines. There is evidence to suggest that adipokines may contribute to the regulation of biological processes, such as metabolism, immunity, and inflammation. The aim of this study was to investigate the usefulness of one of these adipokines in horses, resistin, and its relationship with insulin dysregulation (ID) and inflammation. Seventy-two horses, included in one of the four following groups, were studied: healthy controls, horses with inflammatory conditions, horses with mild, and horses with severe ID. Plasma resistin concentrations were significantly different between groups, and the highest values were recorded in the inflammatory and severe ID groups. The lack of correlation of resistin with basal insulin concentration and the significant correlation of resistin with the inflammatory marker serum amyloid A suggest that, as is the case in humans, plasma resistin concentrations in horses are predominantly related to inflammatory conditions and not to ID.

**Abstract:**

Obesity and its associated complications, such as metabolic syndrome, are an increasing problem in both humans and horses in the developed world. The expression patterns of resistin differ considerably between species. In rodents, resistin is expressed by adipocytes and is related to obesity and ID. In humans, resistin is predominantly produced by inflammatory cells, and resistin concentrations do not reflect the degree of obesity, although they may predict cardiovascular outcomes. The aim of this study was to investigate the usefulness of resistin and its relationship with ID and selected indicators of inflammation in horses. Seventy-two horses, included in one of the four following groups, were studied: healthy controls (C, n = 14), horses with inflammatory conditions (I, n = 21), horses with mild ID (ID1, n = 18), and horses with severe ID (ID2, n = 19). Plasma resistin concentrations were significantly different between groups and the higher values were recorded in the I and ID2 groups (C: 2.38 ± 1.69 ng/mL; I: 6.85 ± 8.38 ng/mL; ID1: 2.41 ± 2.70 ng/mL; ID2: 4.49 ± 3.08 ng/mL). Plasma resistin was not correlated with basal insulin concentrations. A significant (r = 0.336, *p* = 0.002) correlation was found between resistin and serum amyloid A. Our results show that, as is the case in humans, plasma resistin concentrations in horses are predominantly related to inflammatory conditions and not to ID. Horses with severe ID showed an elevation in resistin that may be secondary to the inflammatory status associated with metabolic syndrome.

## 1. Introduction

Obesity and its associated complications, such as metabolic syndrome, are an increasing problem in both humans and horses in the developed world [1]. Equine metabolic syndrome (EMS) is characterized by increased adiposity, insulin dysregulation (ID), and a predisposition to or history of laminitis [2,3,4].

In addition to its energy-storage functions, adipose tissue has endocrine actions mediated by adipokines [5]. Adipokines, which are important in the pathophysiology of obesity and metabolic syndrome [6], are secreted by adipocytes and interact with multiple organs, regulating metabolism, immunity, and inflammation [7,8].

Resistin is an adipokine that was first isolated in mice [9,10]. The place of synthesis and secretion of this hormone is species-specific [9,11]. Elevated concentrations of resistin in plasma have been linked to metabolic changes and inflammation [12]. In humans, resistin is predominantly produced by inflammatory cells, monocytes/macrophages [13], and leucocytes [11,13,14]. Recent evidence suggests that circulating resistin concentrations do not reflect the degree of obesity in humans [15,16,17], but it is related to some inflammatory diseases, such as chronic kidney disease, rheumatoid arthritis, coronary atherosclerosis, T2 diabetes mellitus, and sepsis [18,19]. The relationship between plasma resistin and insulin resistance is not clearly established in humans [9,11,20].

In domestic animals, resistin expression has been demonstrated in fat and mammary tissue from cattle [21] and in leucocytes from pigs [22]. A negative correlation between circulating concentrations of resistin and insulin was noted in cats [23]. An increase in resistin concentrations has been described in cows after calving, and resistin showed a positive correlation with fatty acids [24,25,26]. In dairy goats, resistin has been reported to increase during early lactation [27]. The existence of these relationships suggests that in dairy ruminants, resistin may contribute to the regulation of lipolysis during the peripartum period [24,26]. Very little information is available regarding resistin in horses. Some reports have mentioned resistin as a factor that may represent a link between obesity and insulin dysregulation [9,28]. To our knowledge, there is only one study that has measured resistin in horses [29], and in this study, resistin is correlated with an increase in adiposity.

The aims of the present study were to quantify plasma resistin concentrations and their relationship with related parameters in healthy horses, in horses with metabolic syndrome/insulin dysregulation, and in horses with acute inflammation, in order to evaluate the usefulness of resistin.

## 2. Materials and Methods

### 2.1. Animals

One hundred and eighteen horses were initially enrolled in the study. The population included healthy horses, horses with clinical signs compatible with equine metabolic syndrome (obesity, “good-doers”, divergent hoof rings, and/or regional adiposity), and horses with systemic inflammatory response syndrome (SIRS).

A clinical examination was carried out that included a lameness exam to rule out the presence of active/acute laminitis. The Body Condition Score (BCS) of each horse was assessed using the nine-point Henneke scale [30]. Additionally, local deposition of fat tissue was recorded and the presence of fat deposits in the nuchal crest was assessed using the cresty neck score (CNS) [31]. BCS and CNS scoring were always performed by the same investigator (B.F.).

Peripheral blood samples were collected from the left jugular vein into heparinized tubes. Five to 10 min following the blood collection, tubes were centrifuged at 2500× *g* for 10 min. Plasma samples were stored at −80 °C until measurements were performed.

An oral sugar test (OST) was performed in healthy horses and in horses suspected of having metabolic syndrome. Horses were fasted (no grain) overnight, leaving only access to hay during the previous night. Immediately after obtaining the baseline blood samples (as explained above), corn syrup (Karo Light Corn Syrup; ACH Food Companies, Memphis, TN, USA) was administrated orally by using a 60-mL syringe at a dosage of 0.45 mL/kg of body weight, and blood samples were collected 60 and 90 min after oral dossing [32,33].

After excluding the animals that did not meet the inclusion criteria (horses with active laminitis, pituitary pars intermedia dysfunction, or any disease other than metabolic syndrome or acute inflammation), seventy-two horses (37 males, 27 stallions/10 geldings, and 35 females) aged 3–21 years (Mean ± SD 10, 1 ± 4.87 years) were enrolled in the study. The population included the following breeds: Andalusian (n = 49), Lusitano (n = 6), Crossbreed (n = 11), Oldenburg (n = 2), Thoroughbred (n = 1), Draft horse (n = 1), Welsh (n = 1), and KWPN (n = 1).

Horses were classified in four groups: C = Control group (n = 14): Healthy horses with no signs of equine metabolic syndrome or any other disease in the last 6 months and normal clinical parameters (HR, RR, and temperature) before the OST. These horses had a BCS less than 6.5/9, a basal insulin concentration less than 20 µIU/mL, and a post-glucose insulin concentration less than 90 µIU/mL [3,34]. I = Inflammatory group (n = 21): horses with acute inflammatory conditions and clinical and laboratory signs of SIRS (leukocytosis-leukopenia, tachycardia, tachypnoea, and/or fever). Most of these horses had gastrointestinal inflammatory problems and all of them had SAA > 20 µg/mL. ID = Insulin Dysregulation groups (n = 37): horses with obesity, BCS ≥ than 6.5/9, easy keepers showing regional adiposity, or fat patches, some of them with a history of previous laminitis or divergent hoof rings. All these horses had basal hyperinsulinemia and/or hyperinsulinemia after OST (>90 µU/mL 60 or 90 min after OST) [3,32]. They were subdivided into two groups: ID1 = Mild Insulin Dysregulation (n = 18): basal insulin levels 20–50 µU/mL, and ID2 = Severe Insulin Dysregulation (n = 19): basal insulin levels > 50 µU/mL.

### 2.2. Blood Biochemistry

Plasma concentrations of equine resistin were determined with a commercially available equine resistin ELISA assay kit (RayBio^®^Equine Resistin Elisa Kit) according to the manufacturer’s protocols. The resistin ELISA validation assay showed an intra-assay imprecision of less than 5% and high linearity (r > 0.99) for the measurement of resistin after serial dilutions of a serum sample. The inter-assay CV was <15%. The assay showed a limit of detection (LD) of 2.79 pg/mL. No interferences were observed in hemolyzed or lipemic samples. Plasma concentrations of glucose, iron, total cholesterol, HDL cholesterol, serum amyloid A, and triglycerides were performed in an automated biochemistry analyzer (Olympus AU600, Olympus Diagnostic GmbH1, Hamburg, Germany). All analytes were measured using Beckman kits (Beckman Coulter, Inc., Prague, Czech Republic) with the exception of serum amyloid A, which was measured using a latex agglutination turbidimetric immunoassay (LTIA) Eiken kit (Eiken Chemical Co., Ltd., Tokyo, Japan). Serum insulin concentrations were analyzed using an equine-optimized insulin ELISA (Equine Insulin ELISA, Mercodia AB, Uppsala, Sweden). The insulin ELISA kit showed a sensitivity of 1.15 mU/mL, an intra-assay coefficient < 5%, and inter-assay coefficient < 15%.

### 2.3. Statistical Analysis

Data analysis was performed using SPSS Statistics version 25 software, with *p* values less than 0.05 being considered statistically significant. The data distribution was assessed using the Kolmogorov–Smirnov test. Normally distributed data included iron and HDL cholesterol. One-way ANOVA and Fishers’ LSD post-hoc tests were performed to compare the differences between normally distributed groups. Non-normally distributed data included resistin, glucose, insulin, SAA, triglycerides, and total cholesterol. Kruskal–Wallis and Mann–Whitney Tests were performed on non-normally distributed data. Pearson’s or Spearman’s correlation was used for the analysis of the data sets’ dependency between the plasma resistin concentration and plasma concentrations of the remaining examined blood substances (glucose, insulin, SAA, iron, triglycerides, total cholesterol, and HDL cholesterol) for the entire examined set of horses (n = 72). The correlation coefficient was noted as r with a level of significance less than 5% (*p* < 0.05).

## 3. Results

**Figure 1:**
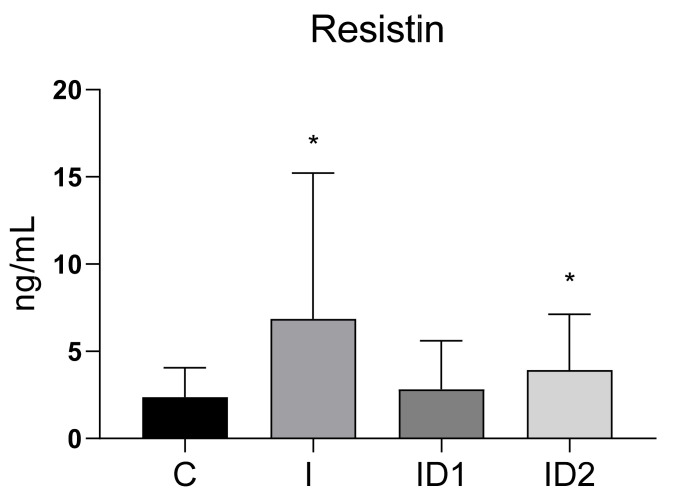
Plasma resistin concentration (ng/mL) in the different groups. Control group (C) n = 14, inflammatory group (I) n = 21, mild insulin dysregulation group (ID1) n = 18, severe insulin dysregulation group (ID2) n = 19. Bars represent mean + SD. Asterisks indicate significant differences with the control group (* *p* < 0.05).

Plasma resistin concentrations in control horses were 2.38 ± 1.69 ng/mL (range 0.07 to 6.47 ng/mL). Resistin concentrations were significantly higher in the inflammatory group at 6.85 ± 8.38 ng/mL (*p* = 0.012) and in horses with severe insulin dysregulation at 4.49 ± 3.08 ng/mL (*p* = 0.035). However, horses with moderate insulin dysregulation did not have increased resistin (2.41 ± 2.70 ng/mL) (Figure 1).

**Figure 2:**
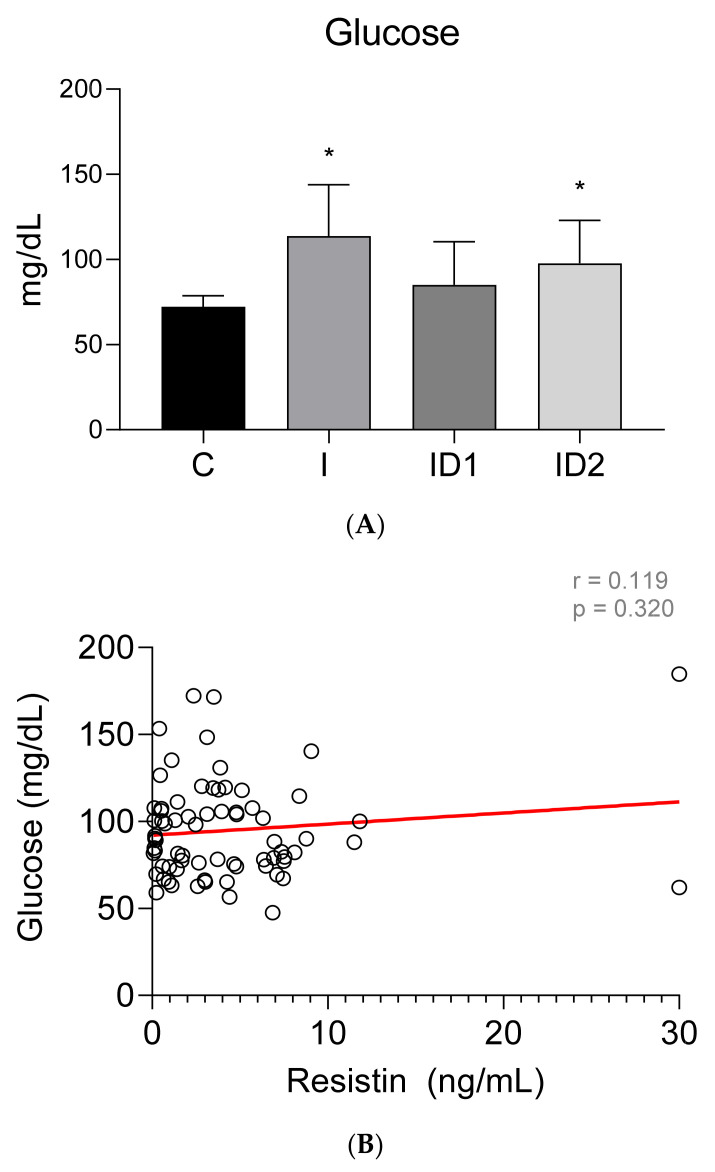
(**A**) Plasma glucose concentration (mg/dL) in the different groups. Control group (C) n = 14, inflammatory group (I) n = 21, mild insulin dysregulation group (ID1) n = 18, severe insulin dysregulation group (ID2) n = 19. Bars represent mean + SD. Asterisks indicate significant differences with the control group (* *p* < 0.05). (**B**) Correlation between plasma glucose concentration (mg/dL) and plasma resistin concentration (ng/mL) in 72 horses. The linear regression line is draw red solid (n = 72, r = 0.119, *p* = 0.320).

Plasma glucose concentrations were significantly increased in horses with inflammatory conditions, 113.80 ± 30.17 mg/dL (*p* < 0.001), and with severe insulin resistance, 99.65 ± 26.07 mg/dL (*p* = 0.002), when compared with healthy horses, 72.30 ± 6.50 mg/dL (*p* < 0.001) (Figure 2A). The plasma glucose concentration in the mild insulin dysregulation group was 85.15 ± 23.74 mg/dL. No significant correlation was found between plasma glucose concentrations and plasma resistin levels (r = 0.119; *p* = 0.320) (Figure 2B).

**Figure 3:**
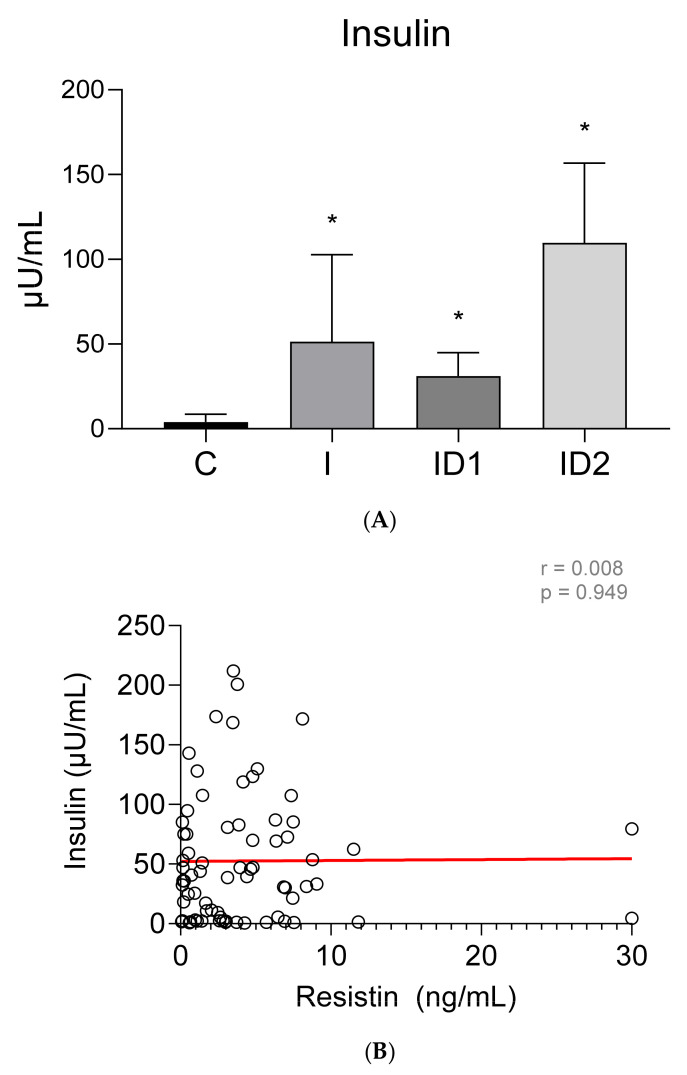
(**A**) Plasma insulin concentration (µU/mL) in the different groups. Control group (C) n = 14, inflammatory group (I) n = 21, mild insulin resistance group (ID1) n = 18, severe insulin dysregulation group (ID2) n = 19. Bars represent mean + SD. Asterisks indicate significant differences with the control group (* *p* < 0.05). (**B**) Correlation between the plasma insulin concentration (µU/mL) and plasma resistin concentration (ng/mL) in 72 horses. The linear regression line is draw red solid (n = 72, r = 0.008, *p* = 0.949).

Plasma insulin concentrations were higher in both groups of insulin dysregulation horses, ID1: 31.16 ± 13.71 µU/mL (*p* = 0.046), and ID2: 109.73 ± 47.04 µU/mL (*p* < 0.001), than in control horses, 3.96 ± 4.66 µU/mL. Horses with inflammatory conditions also showed increased insulin concentrations, 51.45 ± 51.24 µU/mL (*p* < 0.001), (Figure 3A). No significant correlation was found between plasma insulin concentrations and plasma resistin levels (r = 0.008; *p* = 0.949) (Figure 3B).

**Figure 4:**
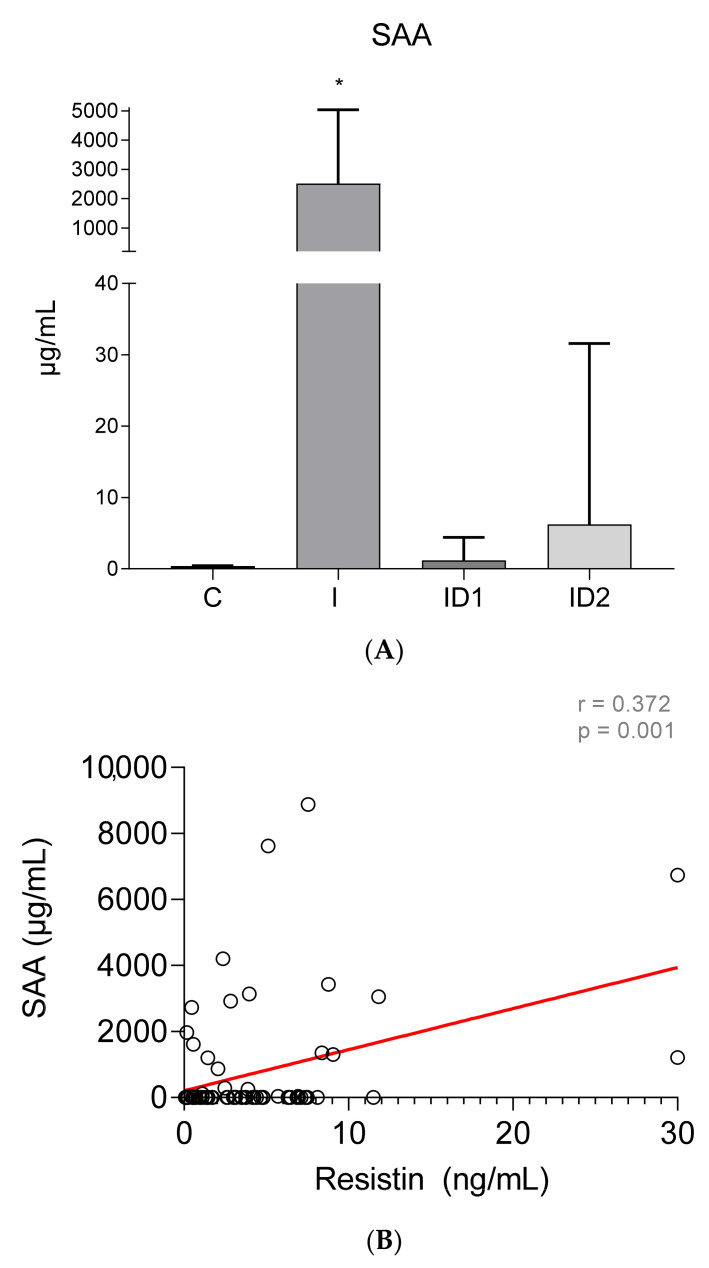
(**A**) Plasma serum amyloid A concentration(µg/mL) in the different groups. Control group (C) n = 14, inflammatory group (I) n = 21, mild insulin dysregulation group (ID1) n = 18, severe insulin dysregulation group (ID2) n = 19. Bars represent mean + SD. Asterisks indicate significant differences with the control group (* *p* < 0.05). (**B**) Correlation between the plasma SAA concentration (µg/mL) and plasma resistin concentration (ng/mL) in 72 horses. The linear regression line is draw red solid (n = 72, r = 0.372, *p* = 0.001).

Concentrations of serum amyloid A (SAA) were very low in control horses, 0.41 ± 0.05 µg/mL. By contrast, horses with inflammatory diseases had extremely high SAA concentrations in plasma, 2518.35 ± 2525.65 µg/mL (*p* < 0.001). Horses with severe insulin dysregulation also had higher SAA, 6.23 ± 25.37 µg/mL than control horses, but those differences were not significant (*p* = 0.555). Horses with mild insulin dysregulation did not have increased SAA, 1.20 ± 3.20 µg/mL (Figure 4A). Significant weak positive correlation was found between plasma SAA concentrations and plasma resistin levels (r = 0.372; *p* = 0.001) (Figure 4B).

**Figure 5:**
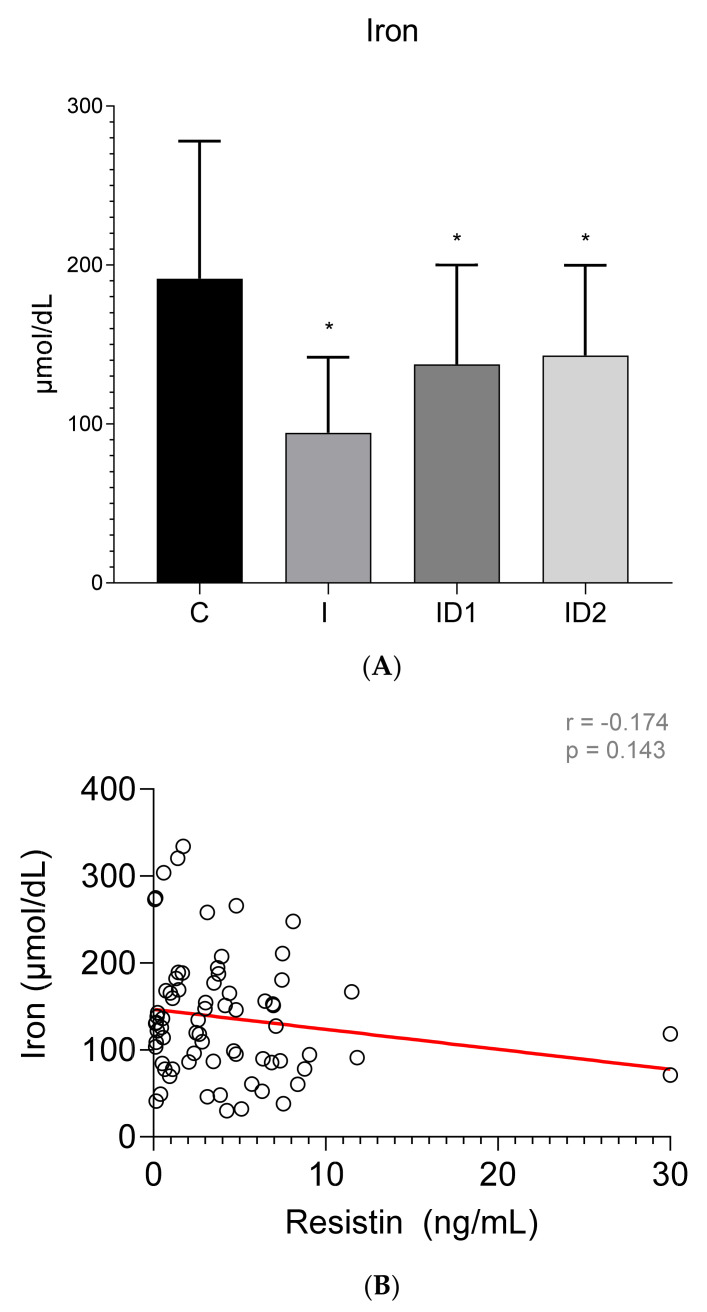
(**A**) Plasma iron concentration (µmol/dL) in the different groups. Control group (C) n = 14, inflammatory group (I) n = 21, mild insulin dysregulation group (ID1) n = 18, severe insulin dysregulation group (ID2) n = 19. Bars represent mean + SD. Asterisks indicate significant differences with the control group (* *p* < 0.05). (**B**) Correlation between plasma iron concentration (µmol/dL) and plasma resistin concentration (ng/mL) in 72 horses. The linear regression line is draw red solid (n = 72, r = 0.174, *p* = 0.143).

Plasma iron concentrations were significantly decreased in the inflammatory group, 94.51 ± 45.58 µmol/dL (*p* < 0.001), and to a lesser extent in both insulin dysregulation groups, 137.51 ± 62.62 (ID1) (*p* = 0.018) and 143.19 ± 57.79 (ID2) µmol/dL (*p* = 0.032), when compared with control horses, 191.50 ± 86.46 µmol/dL (Figure 5A). No significant correlation was found between plasma iron concentrations and plasma resistin levels (r = −0.174; *p* = 0.143) (Figure 5B).

**Figure 6:**
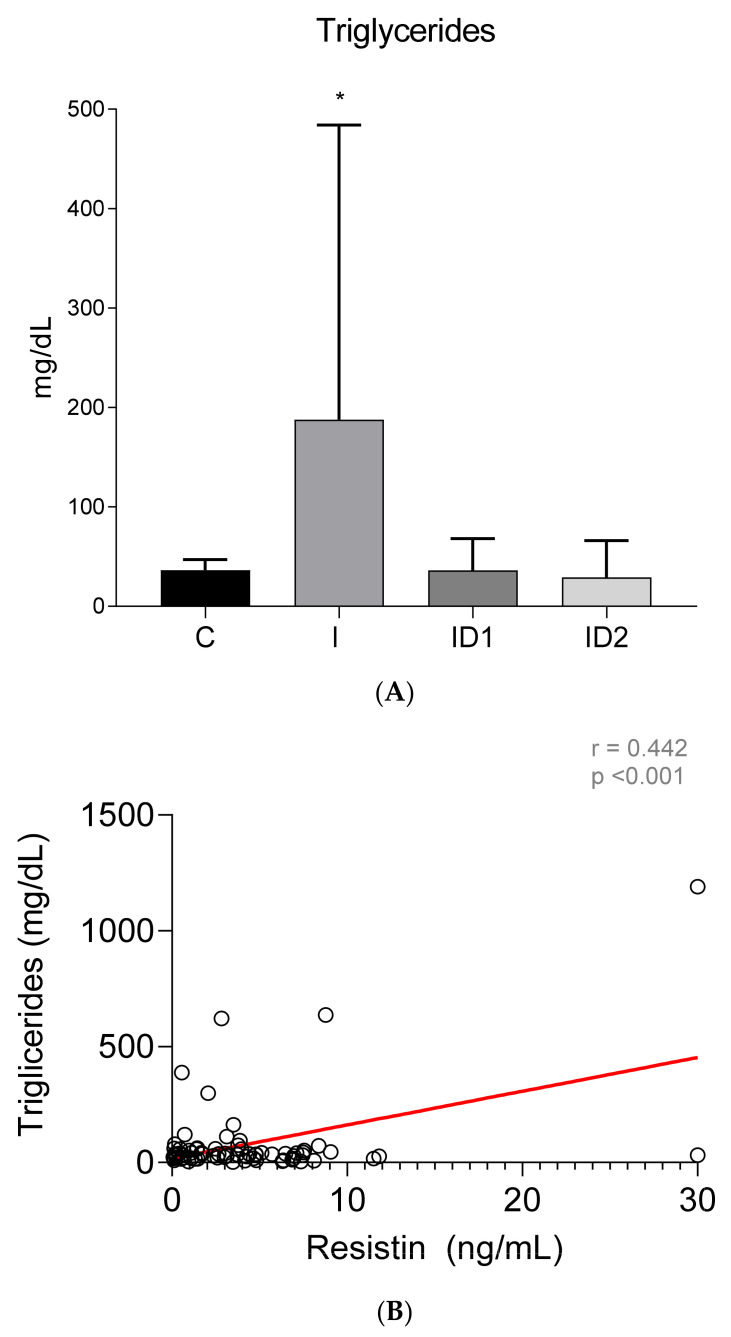
(**A**) Plasma triglyceride concentration (mg/dL) in the different groups. Control group (C) n = 14, inflammatory group (I) n = 21, mild insulin dysregulation group (ID1) n = 18, severe insulin dysregulation group (ID2) n = 19. Bars represent mean + SD. Asterisks indicate significant differences with the control group (* *p* < 0.05). (**B**) Correlation between plasma triglyceride concentration (mg/dL) and plasma resistin concentration (ng/mL) in 72 horses. The linear regression line is draw red solid (n = 72, r = 0.442, *p* < 0.001).

The concentration of plasma triglycerides was significantly higher in horses with inflammatory diseases, 187.61 ± 296.44 mg/dL, when compared with controls, 36.27 ± 10.79 mg/dL (*p* = 0.009). Insulin dysregulation did not influence plasma triglycerides, 36.09 ± 32.10 and 29.17 ± 36.99 mg/dL, in ID1 and ID2, respectively (Figure 6A). A significant average positive correlation was found between plasma triglycerides concentrations and plasma resistin levels (r = 0.442; *p* < 0.001) (Figure 6B).

**Figure 7:**
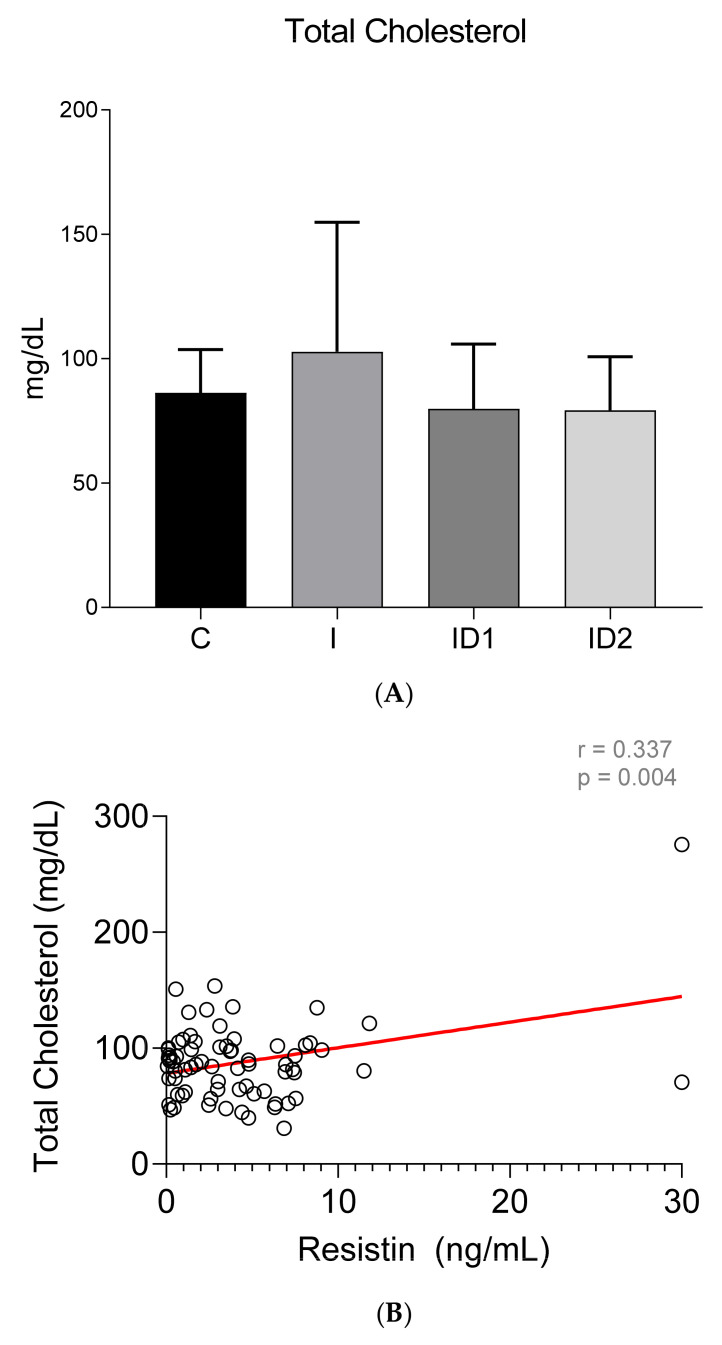
(**A**) Plasma total cholesterol concentration (mg/dL) in the different groups. Control group (C) n = 14, inflammatory group (I) n = 21, mild insulin resistance group (ID1) n = 18, severe insulin resistance group (ID2) n = 19. Bars represent mean + SD. (**B**) Correlation between plasma total cholesterol concentration (mg/dL) and plasma resistin concentration (ng/mL) in 72 horses. The linear regression line is draw red solid (n = 72, r = 0.337, *p* = 0.004).

**Figure 8:**
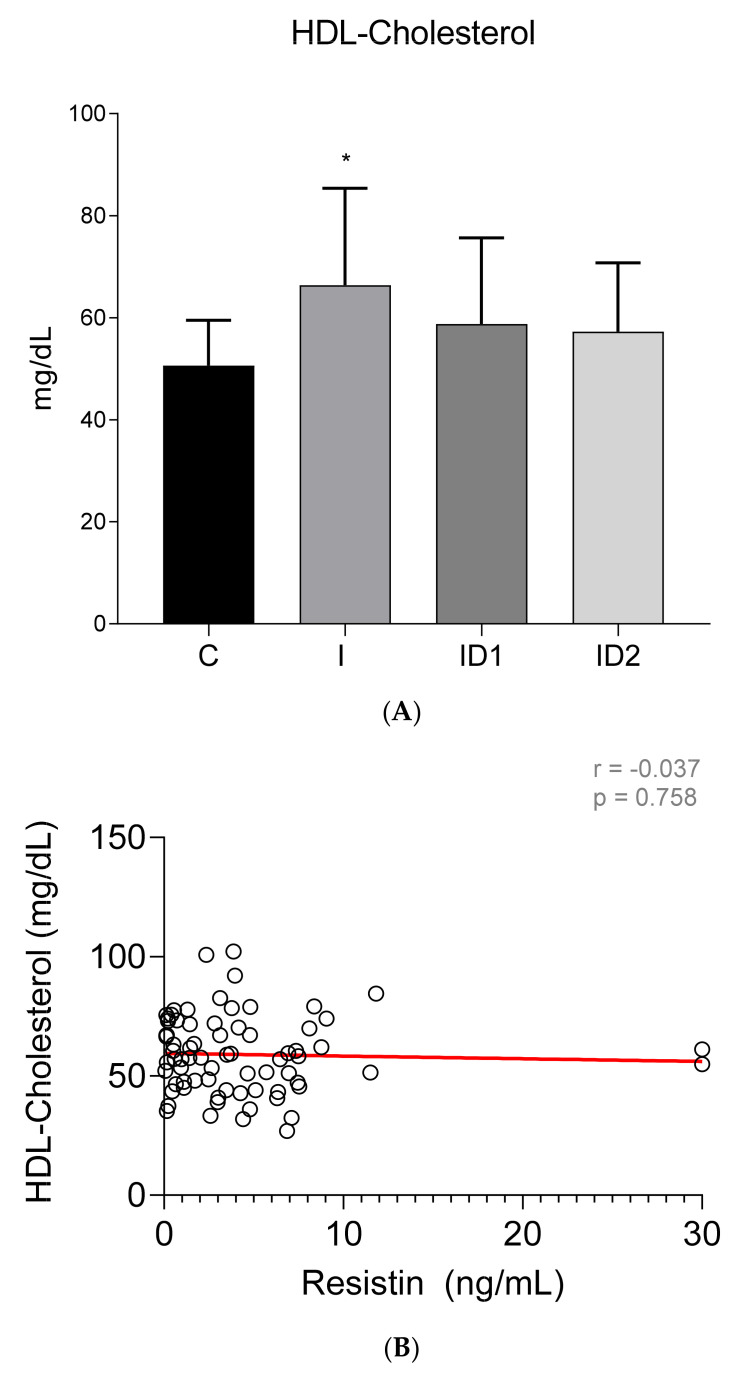
(**A**) Plasma HDL cholesterol concentration (mg/dL) in the different groups. Control group (C) n = 14, inflammatory group (I) n = 21, mild insulin dysregulation group (ID1) n = 18, severe insulin resistance group (ID2) n = 19. Bars represent mean + SD. Asterisks indicate significant differences with the control group (* *p* < 0.05). (**B**) Correlation between plasma HDL cholesterol concentration (mg/dL) and plasma resistin concentration (ng/mL) in 72 horses. The linear regression line is draw red solid (n = 72, r = 0.037, *p* = 0.758).

Although no intergroup differences in total cholesterol concentrations were observed (Control 86.27 ± 17.45 mg/dL; Inflammatory 102.72 ± 52.11 mg/dL; ID1 79.85 ± 29.00 mg/dL; ID2 79.19 ± 21.54 mg/dL) (*p* = 0.108) (Figure 7A), significant week positive correlation was found between plasma total cholesterol concentrations and plasma resistin levels (r = 0.337; *p* = 0.004) (Figure 7B). In contrast with the total values, HDL cholesterol was significantly higher in horses with inflammatory diseases, 66.38 ± 18.98 mg/dL vs. 50.62 ± 8.88 mg/dL in controls (*p* = 0.004) (ID1 58.79 ± 16.87 mg/dL; ID2 57.26 ± 13.49 mg/dL) (Figure 8A) but no significant correlation between HDL cholesterol and resistin was observed (r = −0.037; *p* = 0.758) (Figure 8B).

## 4. Discussion

This study was designed to evaluate the diagnostic value of the measurement of plasma resistin concentrations in horses. Our results demonstrate that resistin can be reliably measured in equine plasma and that plasma resistin concentrations increase preferentially in horses with inflammatory conditions as opposed to horses with insulin dysregulation.

A commercially available ELISA kit for measurement of equine resistin has been used in the present study. The assay demonstrated strong performance for the measurement of plasma resistin concentrations, and our internal validation showed the ability to measure horse resistin with imprecision values lower than 5%, which is considered acceptable for the quantification of biological samples. These results showed a performance similar to a previously used ELISA method [29].

Plasma resistin concentrations in healthy horses (2.38 ± 1.69 ng/mL) were lower than the lower limit of the normal range reported in humans: 7 to 22 ng/mL [35,36,37], although considerable variability has been noted between types of assay. We are only aware of one previous work in which resistin has been measured in equine plasma. Staub et al. [29] reported resistin values in healthy Welsh ponies, which were higher (25.78 ± 4.20 ng/mL) than what we have observed. It is unlikely that the difference is related to breed (ponies vs. horses). The discrepancy is probably related to the different assay used.

The diagnostic value of resistin has been reported to differ between species. While in some species, like rodents, plasma resistin concentrations increase with obesity and metabolic syndrome, in other species, like humans, plasma resistin concentrations increase preferentially when associated with inflammatory problems [38,39,40]. These differences are probably related to the cellular origin of resistin. In mice, resistin is produced mainly by adipocytes, whereas in humans, it is generated predominantly in peripheral blood mononuclear cells, macrophages, and bone marrow cells [13,41,42]. As far as we know, there is still no report on the origin of resistin in horses.

To investigate the diagnostic value of resistin in horses, we studied three categories of animals: healthy controls, horses with inflammatory conditions, and horses with insulin dysregulation/metabolic syndrome. Since metabolic syndrome is also associated with some degree of inflammation, this latter group was divided in two subcategories: moderate and severe insulin dysregulation.

Plasma resistin concentrations were consistently elevated in horses with inflammatory conditions. These results are similar to what has been described in humans [43]. Inflammation is a hyperresistinemic state and several inflammatory diseases, including endotoxemia, rheumatoid arthritis, and inflammatory bowel disease, among others, are associated with increased levels of circulating resistin in humans [7,38,39,43,44,45,46]. Furthermore, serum resistin concentrations are elevated in acute inflammation due to sepsis or systemic inflammatory response syndrome (SIRS) in humans [38]. Our results agree with the data reported regarding humans; thus, the inflammatory group, which included horses with gastrointestinal inflammatory disease and SIRS, had higher levels of resistin.

The mechanisms that influence the elevation of resistin associated with inflammation are not well known. It is believed that resistin may be released by monocytes and macrophages [29,47], and resistin also contributes to the synthesis and secretion of pro-inflammatory cytokines and to differentiation of monocytes into macrophages [35,48]. Furthermore, it has been shown that resistin binds to toll-like receptors (TLR-4) and adenylyl cyclase-associated proteins (CAP-1) to initiate the inflammatory response both in humans and in mice [39,41].

Human studies using an in vitro model have also shown that the mature adipocyte fraction isolated from human adipose tissue is directly involved in both C-reactive protein and serum amyloid A (SAA) release [49]. In our study, changes in SAA paralleled changes in resistin and a significant correlation was found between these two parameters. However, other inflammatory markers used in horses, such as plasma iron concentration, were not correlated with resistin in our study.

Plasma resistin concentrations were not altered in horses with moderate ID but were significantly increased in horses with severe ID. Resistin has been shown to be involved in the control of blood glucose, lipid metabolism, regulation of pituitary somatotropin cells, and the hypothalamic center of satiety [48,50]. However, the role of resistin in obesity and insulin dysregulation is highly controversial [51]. It has been proposed that adipokines may contribute to insulin dysregulation in mice [52], but other studies have argued that the insulin dysregulation mediated by resistin is not reproducible in humans [51,53]. Human studies have shown that individuals with severe insulin resistance have higher resistin levels than healthy individuals [54,55]. However, so far, the mechanisms by which insulin resistance may modulate resistin in humans are not clear.

It is interesting to note that no correlation between resistin and insulin was observed in our study. In a recent meta-analysis regarding the relationship between resistin and circulating levels of insulin resistance, it was concluded that there was no relationship either between circulating resistin and insulin resistance in humans with normal resistin levels. However, “In type 2 diabetes mellitus and obese individuals, resistin levels are positively correlated with insulin resistance in people with hyperresistinemia” [20]. In our study, we have found an elevation in resistin in horses with severe ID, but not in horses with moderate ID. This indicates that, in horses, the relationship between resistin and insulin may be similar to that in humans.

The increase in resistin in horses with severe insulin dysregulation may be related to the inflammatory changes associated with metabolic syndrome. Chronic inflammation is a major and well-known cause of obesity-induced insulin resistance [56], and several pro-inflammatory cytokines play an important role in its pathophysiology. Low-grade chronic inflammation associated with excessive accumulation of adipose tissue and hyperinsulinemia were reported in both humans and horses [57,58,59]. In fact, the main characteristics of equine metabolic syndrome include obesity, insulin resistance, and laminitis, as well as hypertriglyceridemia or dyslipidemia, arterial hypertension, reproductive dysregulation in mares, and an increase in inflammatory markers [3]. In humans, recent studies suggest that resistin directly causes endothelial dysfunction [40] by increasing endotelin-1 (ET-1) secretion as well as vascular cell adhesion protein 1 (VCAM1) and monocyte chemoattractant protein-1 (MCP-1) and by reducing the nitric oxide synthetase [35,50]. Resistin may be helpful in further exploring the connection between vascular disorders in the context of laminitis related to EMS, SIRS, and endotoxemia. In this sense, it would be particularly useful to determine if increased resistin values in a horse with metabolic syndrome may have any predictive value for detecting the risk of laminitis.

Plasma resistin concentrations were also correlated in our study with plasma lipids (triglycerides and cholesterol). The association between resistin and lipids has been previously described in humans, and correlation between resistin levels, fat mass/obesity, HDL cholesterol, and triglycerides, without correlation with insulin dysregulation, has also been described [60,61,62]. Resistin promotes the down-regulation of LDL receptors leading to hypercholesterolemia [63], increases ROS production and LDL accumulation [35,42], and it also promotes elevations in cellular levels of triglycerides and cholesterol [64,65]. In future studies, it would be interesting to evaluate the influence of seasonal mobilization of lipid stores, as described in donkeys [66], on resistin concentrations.

## 5. Conclusions

In conclusion, our results demonstrate that plasma resistin concentrations can be reliably measured in horses. From a diagnostic point of view, the usefulness of resistin seems to be related preferentially with inflammatory conditions and has little value for the diagnosis of insulin dysregulation. Future studies will be needed to better understand the diagnostic value of resistin in a spectrum of inflammatory conditions and to determine if resistin measurements may have diagnostic value to evaluate the inflammatory status in horses with insulin dysregulation.

## Data Availability

The data are not publicity available due to the nature of this work.
